# Effect of neuroactive nutritional supplementation on body weight and composition in growing puppies

**DOI:** 10.1017/jns.2017.57

**Published:** 2017-11-23

**Authors:** Wei Wang, Melissa Brooks, Cari Gardner, Norton Milgram

**Affiliations:** 1 Nestlé Purina Research, One Checkerboard Square, 2RS, St Louis, MO 63164, USA; 2 CanCog Technologies, 120 Carlton Street, Toronto, ON M5A 4K1, Canada

**Keywords:** Nutrition supplementation, Dogs, Canine nutrition, Body composition, Body weight, IGF-1, insulin-like growth factor 1, NNS, neuroactive nutritional supplement, QMR, quantitative magnetic resonance

## Abstract

Nutritional factors can dramatically affect development of young animals during the early stage of life. The objective of the present study was to examine the effects of a neuroactive nutritional supplement (NNS) containing DHA, taurine, carotenoids and vitamins on the body weight and body composition of growing puppies. A total of twenty-four 2-month-old Beagles were fed a nutritionally complete and balanced base diet and a control supplement daily during an initial 1-month baseline assessment, after which they were divided into control and treatment groups. They were fed daily either control or treatment supplements in addition to the base diet from 3 to 12 months of age. Lean body mass and fat mass were assessed using quantitative magnetic resonance scans at 0 (baseline), 3, 6 and 9 months of treatment. Total body weight and lean body mass did not differ between groups over time. The puppies in the treatment group showed a trend of reduced fat gain compared with those in the control group, and with a marginally significant difference at 6 months (*P* = 0·05). At 3 months, insulin-like growth factor 1 was higher (*P* = 0·02) in the treatment group compared with the control group. At 9 months, fasting lipid levels were lower (*P* < 0·05) and fat-oxidation metabolite 3-hydroxybutyrate was higher (*P* < 0·05) in the treatment group compared with the control group. These results may indicate that NNS has an impact on puppy growth and development, possibly by promoting fat metabolism; further investigation would be necessary to determine the full impact of this supplement on growth and development.

The obesity rate has been increasing alarmingly in companion animals, with the dog obesity prevalence rate now estimated to be at 40–50 %^(^^1^^)^. A 2016 survey from the Associations for Pet Obesity Prevention^(^^2^^)^ estimated that 53·9 % of more than 77 million US dogs were either overweight or obese. Canine obesity is of concern because it increases the risk of many diseases, such as diabetes, hypertension, CVD, stroke, dementia and certain cancers.

Weight management by nutritional intervention has typically only been successful through energy restriction, which is an ineffective way of dealing with overweight issues in companion animals, since many pet owners enjoy feeding and giving treats to their dogs as part of the bonding experience. Without careful management of macronutrient diet composition, restriction of food is likely to reduce muscle mass instead of fat mass. Thus, there is a great demand for dietary solutions that can reduce fat gain in companion animals.

The study reported here was part of a puppy growth nutrition intervention study with multiple health benefit outcomes. The nutritional supplement was designed primarily for improving visual function and eye development in growing puppies. The supplement specifically included enhanced levels of DHA (22 : 6*n*-3), taurine, carotenoids and vitamins, and is referred to hereafter as the neuroactive nutritional supplement (NNS).

DHA is an essential fatty acid for the proper development and function of the retina and brain^(^^3^^)^. DHA supplementation in bitches during gestation and lactation has been reported to improve electroretinography (ERG) responses in their puppies^(^^4^^,^^5^^)^. Supplementation with *n*-3 PUFA, including DHA and EPA, has been shown in animal studies to decrease the cellularity of adipose tissue and reduce lipid synthesis^(^^6^^,^^7^^)^. However, supplementation during pregnancy and lactation provided different results from early postnatal supplementation in rat studies^(^^8^^,^^9^^)^. The effects of DHA supplementation on adiposity in growing dogs is, to our knowledge, not currently known.

Taurine is one of the most abundant amino acids in the brain, retina, muscle and other organs. Taurine serves a wide variety of functions in the central nervous system, from development to cytoprotection, and taurine deficiency is associated with developmental abnormalities for the eyes and brain^(^^10^^)^. Taurine is also essential for maintaining normal retinal function and retaining normal ERG function in the cat^(^^11^^)^. There are limited studies on the effect of taurine supplementation on body weight or composition. Taurine supplementation in the late gestation phase stimulated postnatal growth and was associated with increased obesity and insulin resistance in adult offspring^(^^12^^)^. In contrast, taurine supplementation of recently weaned mice fed a high-fat diet reduced obesity and improved glucose homeostasis^(^^12^^,^^13^^)^. As with DHA, these contrasting findings suggest that the timing of supplementation may play a role in the effects seen.

Deficiencies of the carotenoids such as lutein, zeaxanthin and *β*-carotene, and other major antioxidants, such as vitamin C and E, have been associated with increased risks of eye diseases, such as age-related macular degeneration and cataracts^(^^14^^,^^15^^)^. Dietary supplementation of various combinations of carotenoids or lutein/zeaxanthin alone have been shown to preserve or improve visual function in older humans with or without age-related macular degeneration^(^^16^^–^^21^^)^, in healthy younger humans^(^^22^^,^^23^^)^, healthy dogs^(^^24^^)^ and in zebrafish^(^^25^^)^.

To our knowledge, there are no previous reports on the effects of supplementing either *n*-3 PUFA, or taurine, or carotenoids (including specific major carotenoids such as lutein, zeaxanthin and *β*-carotene), or vitamins alone or in combination on the body weight or body composition changes in growing puppies.

The primary target of the nutritional factors included in the NNS is the visual system, with a hypothesis that supplementation would positively influence eye development and visual function. A secondary target is nervous system development, and we hypothesised that cognitive development could also be facilitated by the NNS. Finally, body weight and body composition of the growing puppies were regularly monitored over the course of the project to evaluate the supplement for any beneficial or detrimental effects on growth. The present paper focuses on the body development and body composition portion of the study.

## Materials and methods

### Animals

A total of twenty-four 2-month-old Beagle puppies (twelve males and twelve females) were included in this study. They were followed during their growth period from 2 to 12 months of age. They were either spayed or neutered at about 7 months of age.

During baseline assessment, puppies were housed in groups of six with females and males separated. During the treatment phase, puppies were housed in mixed-sex groups of four with the test group separated from the control group. The puppies were provided with a living space to enjoy group play, exploratory behaviours and running. Environmental enrichment such as toys and social interaction (with penmates and with caregivers) was provided in a consistent manner according to test facility standard operating procedures. In addition, procedures such as leash training, clicker training and cognitive testing provided the dogs with exercise and enrichment outside of their pens in a consistent manner throughout the course of the study. The study protocol was designed in accordance with the principles of the Animals for Research Act of Ontario and the guidelines of the Canadian Council on Animal Care.

Each dog was fed individually to support healthy growth with a commercially available nutritionally complete and balanced diet (Purina ProPlan Chicken and Rice Puppy Formula; Nestlé Purina). Food was offered in portions in accordance with feeding guidelines copied from the original packaging. Portions were adjusted to maintain body condition score close to 3 on a five-point scale^(^^26^^)^, but portions were not allowed to be above or below the feeding guideline ranges recommended by the manufacturer on the bag. Dogs were provided with water *ad libitum*.

The dogs were divided into two groups, matched for age, sex, body weight and cognitive pre-training test ranking. The groups were randomised to receive either a control supplement or the NNS-containing treatment supplement, in addition to the common base diet, during the treatment period when the dogs were from approximately 3 to 12 months of age. The researchers and the caretakers were blinded to the treatment for each group. For the sake of consistency, all references to months throughout the remainder of this paper refer to months on the treatment or control supplement except where noted specifically as age in months.

### Base diet and supplemental foods

All dogs were fed a nutritionally complete and balanced dry dog food formulated for puppy growth (see Table 1) twice daily throughout the 1-month baseline and 9-month treatment study periods.

**Table 1.** Nutrient composition of base diet, supplemental control food and supplemental treatment food for the study*

Nutrient composition	Base diet	Supplemental control	Supplemental treatment
Energy			
kcal/kg diet	5050	4640	4650
kJ/kg diet	21129	19414	19456
Moisture (%)	8·1	8·7	8·3
Protein (%)	30·5	27·9	28·6
Fat (%)	20·2	14·3	15·1
Carbohydrate (%)	33·4	41·3	40·0
Ash (%)	6·3	6·4	6·8
Fibre (%)	1·6	1·5	1·3

AAFCO, American Association of Animal Feed Control Officials.

* The base diet was a nutritionally complete and balanced commercial dog diet meeting AAFCO requirement for puppy growth and development. Both supplemental control and treatment foods were formulated based on a nutritionally complete and balanced commercial dog diet; the treatment food had increased levels of certain nutritional components but with the same energy density. The supplemental foods were fed at 4 g/kg of body weight of the puppies. The amounts were adjusted weekly based on the puppies’ body weight. The total energy to support healthy growth and development was calculated for each dog. The energy supplied by the supplemental foods was subtracted from this calculated total energy need and this remaining energy need was supplied by the base diet.

The energy provided to each dog each day was divided into two feedings; half was given in the morning feeding and the other half in the evening feeding. The supplements were offered first at the morning feeding to ensure that they had been fully consumed before the remaining energy for that feeding were offered as the base diet. The total energy provided to both groups was equal.

The control supplement contained the same amount of energy as in the treatment supplement, with similar macronutrient composition (Table 1). Both supplements were formulated based on the same complete and balanced nutrition formulation with differences in a number of nutritional components, which included higher contents of nutritional factors that promote healthy development. Specifically, the treatment supplement contained higher levels than those of the control supplement of DHA, taurine, the major carotenoids lutein/zeaxanthin and *β*-carotene, and vitamins such as B vitamins, and vitamins A, C, D and E (see Table 2). Each day, 4 g per kg body weight of the supplements were given. The amount of supplements provided was also adjusted weekly based on the dogs’ body weight, which increased as they grew, that is as the dogs’ body weights increased, their daily food supplements were increased accordingly. For example, if a dog's weight was 2 kg, then the dose for the supplement was 8 g; if a dog's weight was 5 kg, then the dose for the supplement was 20 g. A Beagle dog typically grows to a mature body weight of 10 kg; the supplement would therefore be given at 40 g per d.

**Table 2.** Differences in the active nutrient content between groups if fed at 200 g base diet + 40 g of supplemental food*

Nutrients	Unit	Control group	Treatment group	Difference (treatment – control)
DHA	mg	281·12	495·72	214·60
Vitamin A	IU	611·73	699·74	88·01
Vitamin B – niacin	mg	32·84	33·39	0·55
Vitamin B – pantothenic acid	mg	7·08	7·37	0·29
Vitamin C	mg	3·70	83·12	79·42
Vitamin D	IU	1136·33	1224·34	88·01
Vitamin E	mg	34·99	50·30	15·31
Taurine	mg	172·48	444·76	272·28
Lutein/zeaxanthin	mg	11·42	22·31	10·90
*β*-Carotene	mg	0·59	5·73	5·15

* The amounts of various nutrient components in the Table above are the differences between control and treatment supplemental foods for a 10 kg dog (Beagle's mature body weight) with 200 g of base diet and 40 g of supplemental food with supplements fed at 4 g/kg body weight. A commercial nutritionally complete and balanced puppy growth formula (ProPlan Chicken and Rice Puppy Diet) was the base diet. The active nutrient contents were measured using standard analytical methods. The supplemental foods were given at an amount of 4 g/kg body weight daily. The amount was adjusted weekly based on the puppies’ body weight, which changed during growth. As the puppies’ body weights increased, their daily food supplements were increased accordingly. For example, if a puppy's weight was 2 kg, then the dose for the supplemental food was 8 g; if a puppy's weight was 5 kg, then the dose for the supplemental food was 20 g. A Beagle dog typically grows to a mature body weight of 10 kg; therefore, the supplemental food would be given at 40 g per d.

### Body weight and body composition

Body weight and body composition were measured at baseline when the dogs were approximately 3 months of age (at the end of the baseline phase), and subsequently at 3-month intervals of the treatment phase, when the dogs were approximately 6, 9 and 12 months old.

A quantitative magnetic resonance (QMR) (EchoMRI-D QMR analyser; Echo Medical Systems) scan was used to measure body composition by determining the lean body mass and fat mass of the dogs. For the QMR scans, the dogs were placed in a polymethylmethacrylate crate that allowed only limited movement. The crate was placed within the magnet bore of the QMR unit, which was designed for the scanning of animals weighing <50 kg. Dogs were positioned at the magnet isocentre such that their long axis was perpendicular to the long axis of the magnet bore. Data were collected as a standard 3-min fat and water acquisition in accordance with the manufacturer's protocol. This protocol had been previously validated^(^^27^^)^ in awake dogs, which offers the distinct advantage as a non-invasive method to evaluate body composition in growing puppies.

### Blood sampling and analyses

Blood samples were collected at baseline and every 3 months of the treatment phase (3, 6 and 9 months), at the same times body composition measurements were obtained. Blood samples were collected before the morning feeding, with at least an 8 h interval from previous food consumption. Plasma glucose, insulin and insulin-like growth factor 1 (IGF-1) were analysed by the Michigan State University Analytical Laboratory.

Metabolite profiling and lipidomics platform analyses were provided by Metabolon Inc. For the metabolomics analyses, 1618 biochemicals were detected. Metabolite profiling was analysed using ultra-high-performance liquid chromatography/MS (UHPLC/MS) and GC/MS. Fractionation and derivisation of samples and detection technologies have been reported previously^(^^28^^,^^29^^)^. Data extraction, metabolite identification and metabolite quantification were undertaken using proprietary software^(^^28^^,^^29^^)^.

For lipidomics platform analysis, lipids were extracted from plasma in the presence of ^2^H-labelled internal standards using an automated BUME extraction according to the method of Lofgren *et al*.^(^^30^^)^. Each lipid extract was divided between two sample plates, then dried under N_2_ and reconstituted in dichloromethane–methanol containing ammonium acetate. Infusion-MS analysis was performed on a Shimadzu LC with nano PEEK tubing and a Sciex SelexIon-5500 QTRAP. The samples were analysed via both positive- and negative-mode electrospray. The 5500 QTRAP was operated in multiple reaction monitoring (MRM) mode with a total of more than 1100 MRM. Individual lipid species were quantified by taking the ratio of the signal intensity of each target compound to that of its assigned internal standard, then multiplying by the concentration of internal standard added to the sample. Lipid class concentrations were calculated from the sum of all molecular species within a class, and fatty acid compositions were determined by calculating the proportion of each class comprised by individual fatty acids.

While numerous biochemical differences were detected, the present study focuses on the most pronounced differences between the test and control groups. Analyses were conducted on the natural log-transformed data.

### Statistical analysis

Unless specifically mentioned, linear mixed-effects models were run on all of the parameters. To account for the differences between individual puppies, puppy ID was entered as a random effect where the intercept was allowed to vary between puppies. Planned comparisons were conducted on the data looking at differences between the experimental condition *v.* control condition at all four time periods (baseline, 3, 6 and 9 months). For body weight, body composition, insulin, IGF-1, glucose, metabolites and lipids, single-step adjustment comparisons were used to control for the family-wise error rate within each model. For the metabolite and lipid analyses, a different technique was used to control for the family-wise error rate. Because 1618 biochemicals were detected, for each planned comparison we controlled the family-wise error rate across all 1618 biochemicals using the false discovery rate. This type of correction is advisable in situations with large numbers of tests as other methods of family-wise error rate adjustments may be too conservative. In this correction, a *q*-value is calculated which provides an estimate of the proportion of false discoveries for the biochemical which were statistically significant – statistical significance was set at *P* < 0·05^(^^31^^)^. In comparing the test condition *v.* control condition at baseline, there were no statistically significant differences across all of the biochemicals; therefore, the *q*-value was equal to 1. In comparing the test condition *v.* control condition at months 3, 6 and 9 the false discovery rates were as follows: *q*_3 months_ = 0·15; *q*_6 months_ = 0·09; *q*_9 months_ = 0·06. As each parameter has four *P* values – experimental *v.* control at baseline, 3 months, 6 months and 9 months – the notation ‘*P* values’ was used to summarise multiple *P* values. For example, in instances where there was no significant difference at all four time points between the test and control conditions, the smallest *P* value was shown. That is, *P* values > 0·20 indicates that all four of the *P* values were greater than 0·20. Alternatively, *P* values < 0·05 indicates that all four *P* values were less than 0·05. The individual *P* values can be found in the tables.

## Results

Food intake as base diet intake and supplement intake (Table 3) were examined using the linear mixed-effects model previously described. For both food and supplemental intakes, there was no significant difference between groups at all four time points (*P* values > 0·20).

**Table 3.** Food intake, supplement intake, body weight and body composition (Mean values with their standard errors)

	Control		Treatment		
	Mean	se	Mean	se	*P*
Food intake (g)					
Baseline	137·33	1·03	131·17	3·49	0·91
3 Months	193·02	3·99	188·95	5·02	0·98
6 Months	183·62	8·14	184·21	7·93	1·00
9 Months	166·58	7·25	174·79	5·62	0·78
Supplement intake (g)					
Baseline	14·20	0·36	13·30	0·46	0·93
3 Months	33·72	0·92	31·88	1·07	0·56
6 Months	42·84	1·54	39·95	1·38	0·21
9 Months	45·43	1·48	43·17	1·54	0·39
Weight (kg)					
Baseline	3·84	0·09	3·61	0·12	0·91
3 Months	8·56	0·22	8·16	0·26	0·64
6 Months	10·66	0·38	10·05	0·35	0·31
9 Months	11·10	0·34	10·57	0·39	0·42
Lean body mass (kg)					
Baseline	3·01	0·26	3·04	0·35	1·00
3 Months	6·35	0·37	6·25	0·77	0·97
6 Months	7·34	0·67	7·39	0·86	1·00
9 Months	7·61	0·49	7·59	0·83	1·00
Fat mass (kg)					
Baseline	0·68	0·05	0·53	0·03	0·93
3 Months	1·63	0·16	1·30	0·08	0·50
6 Months	2·84	0·27	2·20	0·18	0·05
9 Months	2·86	0·27	2·38	0·25	0·19

When dietary adjustment was necessary to maintain normal body condition score, the base diet amount was reduced approximately 10 % for the specific dog. There were eight dogs in the control group that needed base diet reduction for weight management, with a total of sixteen times for food adjustments. There were four dogs in the treatment group that needed base diet reductions, with a total of eight times for food adjustments during the study. Some dogs’ base diet amounts were adjusted multiple times throughout the study. The relationship between group membership and having at least one food adjustment was examined using a *χ*^2^ test for independence. The relationship between these variables was non-significant (*P* = 0·22).

**Fig. 1.**
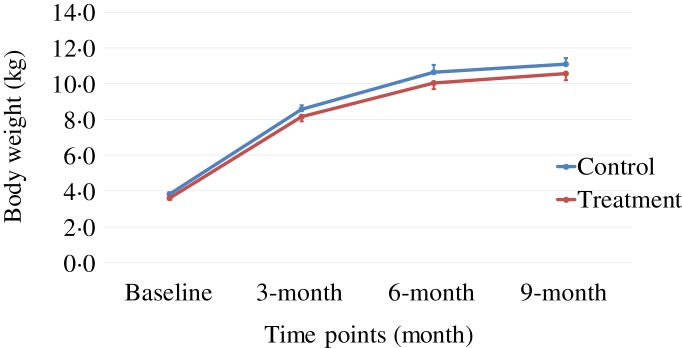
Body weight over time. Values are means, with standard errors represented by vertical bars.

**Fig. 2.**
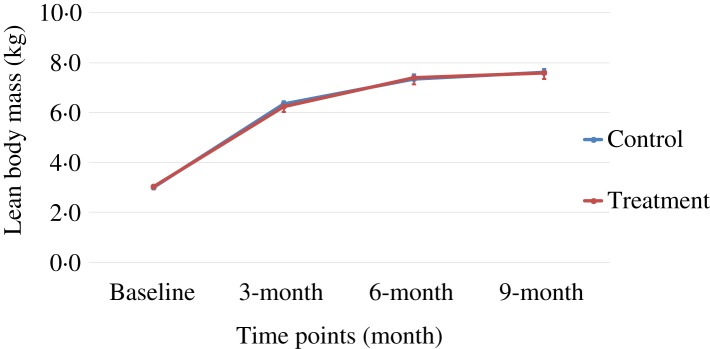
Lean body mass over time. Values are means, with standard errors represented by vertical bars.

**Fig. 3.**
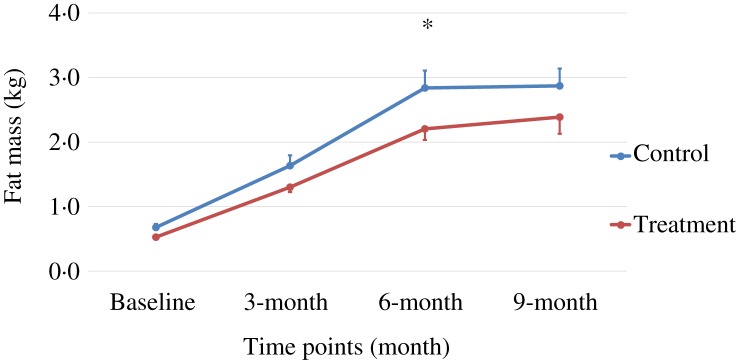
Fat mass over time. Values are means, with standard errors represented by vertical bars. Fat mass gain in the treatment group was trending less (*P* = 0·05) compared with the control group at month 6.

Body weight and body composition measured at baseline (3 months of age) and at months 3, 6 and 9 of supplementation (at 6, 9 and 12 months of age, respectively) were compared between groups. The puppies grew rapidly from 3 to 6 months of age, with their body weights almost doubling during this time period (Table 3 and Fig. 1). The increase in body weight slowed from 6 to 9 months of age, approaching the plateau of the dogs’ mature body weight between 9 and 12 months of age. The body weight changes over time were not different between the two groups (*P* values > 0·31). Lean body mass was not different between groups over time (*P* values > 0·97; Table 3 and Fig. 2). While not statistically significant, the NNS group had lower body fat at every time point; however, this difference was marginally statistically significant at 6 months (*z* = −2·41, *P* = 0·05; Table 3 and Fig. 3).

Descriptive statistics of the relative fold differences between groups (NNS group *v.* control group) in biochemical and lipid parameters at various time points are shown in Table 4. The most obvious biochemical pathway affected by the NNS was lipid metabolism. Total TAG, cholesterol, cholesteryl esters and phospholipids were consistently lower (*P* values < 0·05) in the treatment group compared with the control group at various points in time. In addition, deoxycarnitine, an intermediate in carnitine biosynthesis, was trending higher in the treatment group at the 6 (*P* = 0·06) and 9 (*P* = 0·06) month time points. The final metabolite from fatty acid *β*-oxidation, 3-hydroxybutyrate, was significantly higher in the treatment group compared with the control group at the month 9 time point (*P* = 0·03).

**Table 4.** Biochemical metabolites and lipids from metabolomic analyses* (Normalised mean values with their standard errors)

	Control		Treatment		
	Mean	se	Mean	se	*P*
Cholesterol					
Baseline	0·77	0·05	0·86	0·04	0·15
3 Months	1·09	0·06	1·01	0·05	0·42
6 Months	1·18	0·09	1·03	0·05	0·14
9 Months	1·19	0·11	0·98	0·04	0·05
Cholesteryl esters					
Baseline	0·82	0·03	0·83	0·03	0·76
3 Months	1·08	0·04	1·00	0·03	0·14
6 Months	1·13	0·05	1·02	0·03	0·05
9 Months	1·16	0·06	1·02	0·03	0·02
TAG					
Baseline	1·09	0·08	1·07	0·06	0·91
3 Months	1·05	0·06	0·93	0·05	0·14
6 Months	1·15	0·06	0·91	0·04	0·01
9 Months	1·07	0·05	0·88	0·05	0·02
Phosphatidylethanolamine					
Baseline	1·00	0·04	1·05	0·05	0·36
3 Months	1·07	0·03	0·97	0·04	0·04
6 Months	1·05	0·03	0·95	0·03	0·05
9 Months	1·03	0·02	0·94	0·02	0·07
Lysophosphatidylcholine					
Baseline	0·85	0·03	0·82	0·03	0·42
3 Months	1·05	0·04	0·94	0·02	0·03
6 Months	1·14	0·04	1·01	0·03	0·01
9 Months	1·12	0·04	0·99	0·02	0·02
Lysophosphatidylethanolamine					
Baseline	0·88	0·03	0·92	0·04	0·41
3 Months	1·08	0·05	0·99	0·02	0·10
6 Months	1·08	0·04	1·00	0·03	0·09
9 Months	1·03	0·02	1·01	0·03	0·58
Phosphatidylcholine					
Baseline	0·86	0·03	0·88	0·03	0·44
3 Months	1·04	0·03	0·99	0·03	0·21
6 Months	1·07	0·03	0·98	0·02	0·04
9 Months	1·10	0·03	1·01	0·03	0·04
Phosphatidylinositols					
Baseline	1·05	0·04	1·05	0·03	0·88
3 Months	1·04	0·02	0·98	0·04	0·18
6 Months	1·01	0·03	0·97	0·04	0·32
9 Months	0·95	0·03	0·86	0·04	0·03
Deoxycarnitine					
Baseline	1·04	0·07	0·98	0·07	0·47
3 Months	1·04	0·06	1·07	0·05	0·70
6 Months	0·90	0·04	1·07	0·07	0·06
9 Months	1·01	0·05	1·22	0·10	0·06
Carnitine					
Baseline	1·13	0·05	1·14	0·03	0·89
3 Months	0·84	0·03	0·87	0·04	0·58
6 Months	0·92	0·04	0·93	0·03	0·94
9 Months	1·16	0·05	1·21	0·05	0·43
3-Hydroxybutyrate					
Baseline	1·57	0·19	1·92	0·36	0·44
3 Months	1·16	0·13	1·35	0·18	0·46
6 Months	0·77	0·09	1·09	0·27	0·29
9 Months	0·87	0·19	1·34	0·20	0·03

* While normalised values are shown here, analyses were conducted on the natural log-transformed data.

IGF-1 was significantly higher (*P* = 0·02) at month 3 in the treatment group compared with that of the control group. Glucose and insulin levels were not different between groups over time (*P* values > 0·60; Table 5).

**Table 5.** Insulin, insulin-like growth factor 1 (IGF-1) and glucose for the control and treatment groups during the supplementation study (Mean values with their standard errors)

	Control		Treatment		
	Mean	se	Mean	se	*P*
Insulin (nmol/l)					
Baseline	94·83	9·63	97·33	13·88	1·00
3 Months	111·58	15·50	93·83	9·59	0·61
6 Months	79·50	9·64	71·92	7·08	0·97
9 Months	72·92	8·19	68·67	5·03	1·00
IGF-1 (nmol/l)					
Baseline	20·08	1·54	20·17	1·79	0·97
3 Months	22·50	2·05	29·08	2·26	0·02
6 Months	16·25	1·15	18·58	1·09	0·42
9 Months	14·25	1·50	16·92	1·26	0·42
Glucose (nmol/l)					
Baseline	6·49	0·08	6·42	0·11	0·97
3 Months	5·96	0·11	5·98	0·11	1·00
6 Months	5·83	0·08	5·66	0·12	0·64
9 Months	5·98	0·11	5·70	0·09	0·17

## Discussion

The growth pattern of body weight observed in this study was similar to that which had been reported by others^(^^32^^)^, in which male Beagle puppies were shown to approach their mature body weights at about 28–31 weeks (approximately 7–8 months) of age. Similarly in our study, Beagle puppies were reaching a plateau of body weights between 24 and 36 weeks of age. All puppies grew and gained weight at rates that fell within the margin of error of previously established puppy growth curves^(^^32^^)^.

The impacts of birth weight and litter size were not measured in this study. The birth weights of the puppies were not different between the groups (*P* = 0·87). Of the twenty-four puppies in the study, fourteen were singles from genetically unrelated litters, and ten were male/female pairs from five genetically unrelated litters. The randomisation did not account for litter pairs; as a result, two litter pairs were split between the treatment groups and three litter pairs were in the treatment group. This presents a potential confounding factor. However, all puppies were healthy and their weights were consistent with validated growth curves. In addition, none of these puppies became overweight or obese from the study. Research on human subjects has shown that low birth weight may increase the risk of obesity and diabetes^(^^33^^)^; further investigation is needed to determine if this occurs in dogs. However, no significant relationship was found between birth weight and weight at the end of this study (*P* = 0·27).

The treatment group showed a non-significant trend of lower levels of fat mass at all time points, although it was marginally statistically lower at 6 months (*P* = 0·05), while the lean body mass gain was the same compared with those of the control group during this growth study (Table 3, Figs 1–3). It is commonly known that after spaying or neutering, dogs may gain weight, which is mainly from body fat gain^(^^34^^)^. In our study, the dogs were neutered or spayed at about 7 months of age. Although the changes in average total body weight for the two groups were not statistically significant during the study period, the body fat gain in the time period from 6 to 9 months (9 to 12 months of age) was less in the treatment group compared with the control group (Fig. 3). This suggests that nutritional intervention may help to offset the fat gain associated with neutering or spaying.

In addition to the trend of reduced fat mass accumulation in the treatment group, the treatment dogs required fewer adjustments to their food amounts to maintain ideal body condition; four dogs in the treatment group required a total of eight adjustments, while eight dogs in the control group required a total of sixteen adjustments. While the number of dogs requiring adjustments was not statistically significantly different between groups, a larger sample size with greater power may provide more insight into the role of supplementation in weight management.

In a rat study, *n*-3 PUFA supplementation during pregnancy and lactation resulted in an increased percentage of subcutaneous fat in offspring^(^^8^^)^. *n*-3 PUFA supplementation in early postnatal life in rats reduced fat accumulation, improved lipid and glucose homeostasis, and was associated with fewer hypertrophic adipocytes in adulthood. Supplemented rats also exhibited lower absolute and relative fat mass compared with controls, with no significant changes in lean body mass between treated and control subjects^(^^9^^)^. These conflicting results in rat studies raise the possibility that nutritional influence during different phases of perinatal development (prenatal compared with early postnatal period) might confer different susceptibilities toward increased fat storage, and highlight the need for further studies. Note that critical periods (e.g. prenatal compared with early postnatal) might differ among species, and thus findings from rodent studies or human studies cannot be generalised to dogs^(^^9^^,^^35^^)^. Our study showed that puppies receiving the NNS tended toward reduced fat accumulation with preserved lean body mass compared with controls, similar to observations by Oosting *et al*.^(^^9^^)^. Because our study period ended at 1 year of age, and without overweight or obese conditions observed in any of the puppies, conclusions regarding the impact of NNS supplementation on obesity and lipid and glucose metabolism in adulthood cannot be made.

While maternal taurine supplementation in the late pregnant rat stimulated postnatal growth and induced obesity and insulin resistance in adult offspring^(^^12^^)^, taurine supplementation of recently weaned mice fed a high-fat diet attenuated obesity and improved glucose homeostasis as well as a reduction in the hypothalamic content of phosphorylated insulin receptor substrate 1, a critical component of insulin-signalling pathways; this mechanism was believed to be linked to the prevention of overfeeding, glucose intolerance and obesity^(^^13^^)^. It is possible that the taurine component of the NNS contributed to the findings observed in the present study; however, because the NNS was tested as a blend of ingredients, the impact of individual components is unknown.

It has been postulated that an excess of adipocytes results in both an increased predisposition toward obesity in adulthood and an increased difficulty in maintaining weight loss when it occurs. This theory has been supported by several studies on laboratory animals showing that early overnutrition results in increased numbers of fat cells and increased total body fat throughout adult life^(^^36^^,^^37^^)^. Therefore, it is reasonable to postulate that the puppies in the supplemented group with reduced body fat mass accumulation during early development may have fewer adipocytes and, therefore, a better chance of maintaining a healthy body weight and body composition later in life; however, this hypothesis was not tested in our study. Because the present study only evaluated the dogs over the first year of life, the effects of longer-term NNS supplementation on body composition are unknown. The development of supplements that support the preservation of lean body mass while reducing, or preventing the accumulation of, fat mass may be particularly useful for dog breeds that are prone to obesity such as Basset hound and Beagle.

The prevalence of overweight or obesity in 1-year-old dogs has not been investigated or reported as robustly as the prevalence in older dogs. A multi-centre study of the medical records of more than 21 000 dogs aged 1 year or above determined a rate of 13·1 % overweight and 1·1 % obesity in 1-year-old dogs in the USA^(^^38^^)^. An Australian survey-based study of veterinary assessments of 2661 dogs determined a rate of 18·6 % overweight and 2·4 % obesity^(^^39^^)^. However, more recent data are lacking. None of the puppies in this study was overweight or obese at the conclusion of the study, but this was attributed to consistent monitoring and adjustment of food amounts in order to maintain optimal body condition scores. Although body condition scoring was utilised by trained technicians to determine if food adjustments were indicated, it was not recorded as a parameter for statistical analysis.

Blood results were in agreement with the body development differences observed in the study. IGF-1 levels were significantly higher at month 3 (*P* = 0·02). IGF-1 is known to play important roles in normal growth and development, and is specifically known for promoting the growth and repair of skeletal muscle^(^^40^^)^. The levels of IGF-1 in the growing puppies observed in this study were similar to those previously reported in dogs^(^^41^^–^^43^^)^. The higher level of IGF-1 in the treatment group compared with that of the control group was consistent with the biological function of IGF-1 to facilitate use of glucose and amino acids for building muscle, shifting the body toward utilisation of fat as an energy source and contributing to less fat accumulation over time.

IGF-1 is the primary mediator of the physiological effects of growth hormone. IGF-1 levels are used by physicians as a screening test for growth hormone deficiency or excess, as IGF-1 levels – unlike growth hormone levels – do not fluctuate greatly throughout the day for an individual. The higher IGF-1 levels found in the puppies who received the NNS in this study are possibly a result of a higher growth hormone level secretion, thus promoting growth.

Growth hormone has also been reported to have a favourable impact on obesity management. Human subjects with abdominal obesity who were treated with growth hormone for 9 months were reported to have 9 % decreases in total body fat, decreases in cholesterol, TAG and diastolic blood pressure, and an increased glucose disposal rate^(^^44^^)^. The lower body fat gain in the treatment group of the dogs in the present study could be a result of a combined effect from the higher level of growth hormone in conjunction with the higher IGF-1 levels.

Metabolomics analyses showed that fasting circulating lipids were lower in the treatment group than those of the control group. The trending increase of deoxycarnitine in the treatment group also indicated increased *de novo* carnitine biosynthesis to support increased fatty acid *β*-oxidation. Aligning with this metabolic path, the level of 3-hydroxybutyrate was significantly higher in the treatment group at month 9, indicating that dogs in the treatment group were using fatty acids at a greater rate than control dogs.

We observed lowered circulating lipids, increased fat oxidation and increased IGF-1 in the treatment group compared with the control group. This suggests that energy metabolism was shifted from storage (as fat mass) toward utilisation for energy. A biochemical profile such as this favours a healthier metabolic status for the growing puppies. There were no significant differences in blood glucose or insulin levels between the control and treatment groups that would suggest a negative energy balance, and all puppies gained weight consistently with established growth curves; therefore, the shift toward fat utilisation was not driven by energy deprivation.

Because the components of the NNS were not assessed individually for their effects on growth or body composition, the authors are unable to speculate which ingredient or combination of ingredients affected the metabolic alterations observed in this study population. Further investigation is needed to understand which of the tested nutritional factors or maybe a synergy of all of them play a role on body development and metabolism in puppies.

To the authors’ knowledge, this is the first report of the use of quantitative MRI to accurately, rapidly and safely assess body composition of puppies. The procedure allows for the non-invasive measurement of body composition without the need for sedation^(^^27^^)^.

In summary, optimum nutrition is essential for healthy early development, including healthy body development, of an animal. Formulations to improve body composition towards less fat and leaner body mass for growing dogs are limited. A supplemental food form as used in the present study is a practical nutritional intervention strategy. This study showed that optimising nutrition with the NNS formulated in this study not only resulted in a trend toward less fat mass gain but also in an overall healthier metabolic profile in the development of puppies. This suggests that even though the puppies in this study received a nutritionally complete and balanced base diet based on American Association of Animal Feed Control Officials (AAFCO) standards^(^^45^^)^, the optimised nutritional supplementation could shift the puppies’ body development towards a leaner body composition. The leaner body composition and healthier metabolic profile may establish a foundation for puppies to have better body weight management later in life.
